# Comment on Clayton-Chubb et al. Understanding NAFLD: From Case Identification to Interventions, Outcomes, and Future Perspectives. *Nutrients* 2023, *15*, 687

**DOI:** 10.3390/nu15132907

**Published:** 2023-06-27

**Authors:** Ludovico Abenavoli, Giuseppe La Torre, Natasa Milic

**Affiliations:** 1 Department of Health Sciences, University “Magna Græcia”, Viale Europa—Germaneto, 88100 Catanzaro, Italy; 2 Department of Pharmacy, University of Novi Sad, 21000 Novi Sad, Serbia

This letter is to comment on the article by Clayton-Chubb et al. on the pathogenesis of non-alcoholic fatty liver disease and its outcomes, and the description of current, emerging and future directions to treat this condition.

We read with great interest the recent review by Clayton-Chubb et al. on the treatment options in patients with non-alcoholic fatty liver disease (NAFLD) [1]. NAFLD is a global pandemic which affects around 25% of the worldwide population, characterized by the accumulation of fat in the liver, due to a multistep process [2]. The literature has described molecular, biochemical, and biophysical abnormalities in the NAFLD context [3]. Several studies on the mechanisms involved in the onset and development of NAFLD highlight the role of genetic polymorphisms in enhancing oxidative stress, pro-inflammatory cytokines production, and in the imbalance of glucose and lipid metabolism [4]. In addition, epidemiological studies have shown that NAFLD is associated with increases in all-cause and liver-related mortality compared with the general population [5]. The standard of care to treat NAFLD, as described by international guidelines, is focused on lifestyle changes and, in particular, on starting a healthy diet and increasing physical exercise [6]. However, there are no currently licensed pharmacological treatments for NAFLD, except lifestyle modification by diet and exercise. Phosphatidylcholine is one of the drugs under investigation by the scientific community, characterized by a significant positive anti-oxidative and anti-fibrotic effect on NAFLD [7]. In particular, in a recent randomized controlled trial and meta-analyses, 3-sn-phosphatidylcholine showed a regression of steatosis [8,9]. However, despite different evidence, the mechanisms of action of phosphatidylcholine are still poorly understood. In this context, we have recently evaluated the effects of 3-sn-phosphatidylcholine supplementation, at a dose of 600 mg three times daily for three months, in obese patients with NAFLD [10]. Our results indicate that the treatment with the administration of phosphatidylcholine, was associated not only with a significant improvement in the biochemical hepatic profile of transaminases, but also with the increase in the blood levels of antioxidant enzymes such as superoxide dismutase and glutathione peroxidase. These results are in line with a previous study by our group, where we describe the efficacy of phosphatidylcholine associated with silybin and vitamin E, to induce a significant statistical improvement in anthropometric parameters, lipid and glucose profiles, and intra-hepatic fat accumulation [11]. According to the data of Clayton-Chubb et al., we can conclude that new therapeutic targets are now under investigation for NAFLD. In this context, on the basis of our pilot study and considering the limited side effects registered, we support the possible role of phosphatidylcholine in association with lifestyle changes, to treat patients with NAFLD. However, a large randomized clinical trial to confirm our data is needed.
